# Editorial: Editors’ showcase 2024: insights in morphogenesis and patterning

**DOI:** 10.3389/fcell.2025.1712557

**Published:** 2025-10-06

**Authors:** Glenn S. Edwards, Shinji Takada

**Affiliations:** ^1^ Physics Department, Duke University, Durham, United States; ^2^ National Institute for Basic Biology, Okazaki, Japan

**Keywords:** morphogenesis, patterning, development, zygotic activation, evolution

Edward Lewis, Christiane Nusslein-Volhard, and Eric Wieschaus shared the 1995 Nobel Prize in Physiology or Medicine for their research on the genetic control of early development (Lewis, 1978; Nusslein-Volhard and Wieschaus, 1980). They described how maternal factors trigger a genetic cascade that directs the embryo’s body plan through activation of the spatial and temporal expression of zygotic genes. The genes involved in this genetic cascade were subsequently shown to have been conserved for millions of years of evolution and thus to apply broadly to the development of biological organisms.

This Focus Research Topic highlights research that further advances our understanding of the mechanisms of morphogenesis and pattern formation during embryonic and tissue development. In addition to sophisticated molecular genetic methodologies, advances in imaging technology, the use of omics data, and the analysis from a physical perspective have led to substantial contributions. We aim to celebrate recent discoveries and novel insights, to identify current challenges, and to consider future perspectives in this field of research.

This Research Topic includes two review papers. One review discusses the mechanisms that generate left-right asymmetry in the body. In many vertebrate model organisms left-right asymmetry of the body is established based on the unidirectional rotation of the nodal cilia, but it remains unclear how left-right asymmetry is broken in chickens where nodal cilia do not rotate. Here, Pieper and Tsikolia present their perspective on the mechanism for left-right symmetry breaking in the chicken. In addition, Li et al. review the role of the Nuclear receptor subfamily 6 group A number 1 (Nr6a1), which is an orphan nuclear receptor that has no close evolutionary homologs. This review provides a comprehensive introduction of Nr6a1, including: its dynamic expression pattern in development; its regulatory mechanisms of expression; its roles in development; its molecular mechanisms and targets; and a consideration of its potential as a molecular target for the evolution of the animal body plan.

This Research Topic also includes five papers that report original research using a wide range of animal models. During the past decade, it has become clear that the metabolic state of cells has a significant impact on cell differentiation. Using cellular slime molds, *Dictyostelium discoideum*, Abe et al. suggested a strong link between glucose metabolism and cell differentiation. Under starvation *D. discoideum* cells aggregate and differentiate into two major cell types, prestalk and prespore cells. Here, the authors provide evidence that cell fate determination between these two cell types is controlled by the levels of phosphoenolpyruvate carboxylase (PEPC), a key enzyme that determines metabolic flux in glycolysis, the TCA cycle, and gluconeogenesis.

Intercellular signalling plays an important role in the morphogenesis and patterning of multicellular organisms. In recent years, numerous studies have shown that specialized cell processes are involved in intercellular signalling. Bowman et al. previously reported that cell processes called airineme are involved in Notch signalling during pigment formation in zebrafish. An early step in airway-mediated signalling is the engulfment of blebs at the tip of the airineme by macrophages. Here, the authors demonstrate that the extracellular domain of the membrane protein CD44 is involved in this process.

The vertebrate neural tube is widely used as a model for patterning, as various types of neurons arise along the dorsal-ventral axis. However, the morphology of the neural tube changes during later stages of development, with the roof plate (a signaling center for patterning) undergoing significant changes in shape. Shinozuka et al. have previously shown that Wnt signaling is required for this roof plate’s morphological change, and here they suggest that Wnt-dependent apical constriction of roof plate cells may be involved.

Accumulation of precise cell lineage analyses and molecular genetic studies have revealed that the differentiation of vertebrate neural cells is closely related to the differentiation of cells of other lineages. Otx2 is widely known as one of the key factors in vertebrate nervous system development, but its role in early nervous system development remains unclear. To answer this question, Takami et al. investigated the effects of ectopic expression of Otx2 in the epiblast of chick embryos, where undifferentiated neural cells exist during embryonic development.

During early tetrapod development each vertebra gains a distinctive structure, where the location of limb development depends on position along the anterior-posterior axis. Saito et al. previously reported that Gdf11 determines the position of the sacral and hind limbs, and here they focus on how spatial expression of gdf11 is regulated. As a result, they found that a highly conserved region within the gdf11 intron and FGFs are independently involved in the regulation of gdf11 expression, indicating the existence of a complex regulatory mechanism.

This Research Topic also includes one paper that reports hypothetical and theoretical research. Koyama et al. previously published an image processing method to estimate intercellular mechanical forces from live images of multicellular tissues. Here, they report an extension of that method to accommodate cavity-harbouring tissues and aimed to validate this extended method with the analysis of mouse blastocytes images.

Decades have passed since the foundational research of Lewis, Nusslein-Volhard, and Wieschaus. As described here and elsewhere, the field of morphogenesis and patterning is vibrant, in pursuit of answers to the many questions that address how genetics coopts chemistry and physics during the patterning processes of morphogenesis and development.
